# Total knee arthroplasty exhibits satisfactory long-term clinical efficacy in the treatment of hemophilia patients with stiff knees

**DOI:** 10.3389/fsurg.2022.1014844

**Published:** 2023-01-06

**Authors:** Yi Liu, Yi-fan Liu, Hong-zheng Meng, Tao Sun, Ping Gao, Zhao-zhi Li, Wen-qiang Zhang

**Affiliations:** ^1^ Postgraduate Department, Shandong First Medical University, Jinan, China; ^2^ Department of Orthopaedic Surgery, The First Affiliated Hospital of Shandong First Medical University & Shandong Provincial Qianfoshan Hospital, Shandong Key Laboratory of Rheumatic Disease and Translational Medicine, Jinan, China

**Keywords:** hemophilia, stiff knee, total knee arthroplasty, outcomes, complication, survival

## Abstract

**Objective:**

This study aimed to (1) determine the long-term clinical efficacy of total knee arthroplasty (TKA) in the treatment of hemophilia patients with stiff knessknees, (2) assess the 5- and 10-year prosthesis survival in hemophilia, and (3) determine whether the severity of preoperative stiffness would affect postoperative clinical outcomes and complication rates.

**Methods:**

The clinical data of 71 patients (78 knees) with hemophilia and concomitant knee stiffness who had undergone TKA between September 2007 and June 2018 were retrospectively analyzed. All patients were male, their mean age at the time of surgery was 38.4. ± 7.9 years (range: 21–63 years), and the mean follow-up time was 8.7 years. To determine the effect of stiffness severity on clinical outcomes, the participants were categorized into two groups: severe [preoperative range of motion (ROM): <50°, 34 knees] and moderate (preoperative ROM: 50–90°, 44 knees). At preoperative and final follow-up, patients' post-TKA clinical and radiological outcomes, quality of life, complications, and long-term survival were assessed.

**Results:**

Flexion contracture improved from 23.2 ± 10.8° before surgery to 5.9 ± 7.5° upon final follow-up, the Knee Society Score (KSS) increased from 31.4 ± 12.4 to 74.9 ± 11.5, and the KSS functional score increased from 37.6 ± 9.3 to 81.4 ± 12.8. The mean ROM improved from 54.6 ± 32.6° preoperatively to 80.9 ± 34.5° postoperatively. The 36-Item Short Form Survey physical and mental scores also improved significantly. All these differences were statistically significant before and after surgery (*P* < 0.001). The following postoperative complications occurred in 10 knees (12.8%): hemarthrosis (*n* = 3), stiffness (*n* = 4), superficial infection (*n* = 1), skin necrosis (*n* = 1), and periprosthetic infection (*n* = 2), and revision TKA was performed on two knees. The 5- and 10-year survival rates of the prostheses were 98.5% and 93.7%, respectively. The mean ROM in the severe group increased from 30.7 ± 18.7° preoperatively to 70.5 ± 28.3° postoperatively (*p* < 0.001). The mean flexion contracture decreased from 27.3 ± 10.8° to 6.4 ± 12.0° (*p* < 0.001). The mean KSS improved from 27.0 ± 7.8 to 68.3 ± 9.6 (*p* < 0.001). The mean ROM in the moderate group improved from 84.3 ± 22.7 to 92.9 ± 28.8 (*p* < 0.001), while the mean flexion contracture decreased from 12.8 ± 11.0° to 4.8 ± 5.0° (*p* < 0.001) and the mean KSS improved from 41.3 ± 11.5 to 81.3 ± 12.2 (*p* < 0.001). The severe group had worse postoperative ROM and functional scores than the moderate group. Furthermore, the severe group used varus-valgus constrained or hinged prostheses more frequently (52.8% vs. 18.1%) and had more complications (18.9% vs. 9.0%) than the moderate group.

**Conclusion:**

TKA exhibits satisfactory long-term efficacy in patients with hemophilic knee joint disease involving preoperative stiffness, thus potentially providing a significant improvement in function and reducing pain. Furthermore, severely stiff knee joints have worse clinical outcomes and more complications than moderately stiff knee joints.

## Introduction

Hemophilia is a hereditary bleeding disorder caused by a deficiency of clotting factors. Hemophilia A is the most common type of the disease and often manifests as repeated spontaneous bleeding in the joints and muscles, predominantly occurring in the knee, elbow, and ankle joints. Repeated intra-articular hemorrhage leads to hemosiderin deposition; synovial hyperplasia; articular cartilage destruction; and eventually joint pain, deformity, and dysfunction, which seriously affect patients' quality of life (1).

Owing to repeated bleeding and swelling of the knee joint, patients are forced to adopt the knee joint flexion position, resulting in knee joint flexion deformity, joint stiffness, and difficulty in surgical treatment. TKA in patients with hemophilia remains challenging to treat, with failure rates as high as 20% having been reported in the literature (2). Preoperative range of motion (ROM) is usually considered to be the most important predictor of functional outcome after TKA. Preoperative knee stiffness (defined as a preoperative ROM of 50 or less) has been shown to significantly impair overall clinical outcomes (3). In addition, a higher complication rate due to difficulty in knee exposure, soft tissue balance, and iatrogenic injury to extensors or patellar tendons has been reported (4).

Several studies involving short-to-medium-term follow-up have demonstrated that TKA for hemophilic knee arthritis can effectively relieve pain and improve joint mobility and patient satisfaction. However, the post-TKA prosthetic survival rate for patients with hemophilia is reported to be lower than that of patients without hemophilia, such as older patients with primary osteoarthritis (5–7). Possible reasons include younger age at the time of surgery, higher activity, heavy wear of the prosthesis, and higher incidence of postoperative infection. To date, studies have included only a few hemophilia cohorts with postoperative long-term TKA performed at multiple centers, and there are few available data reporting long-term outcomes in hemophilia patients. Therefore, this study aimed to (1) determine the long-term clinical efficacy of TKA in the treatment of hemophilia patients with stiff knees, (2) assess the 5- and 10-year prosthesis survival in hemophilia, and (3) determine whether the severity of preoperative stiffness would affect postoperative clinical outcomes and complication rates.

## Materials and methods

### Inclusion and exclusion criteria

**Figure 1:**
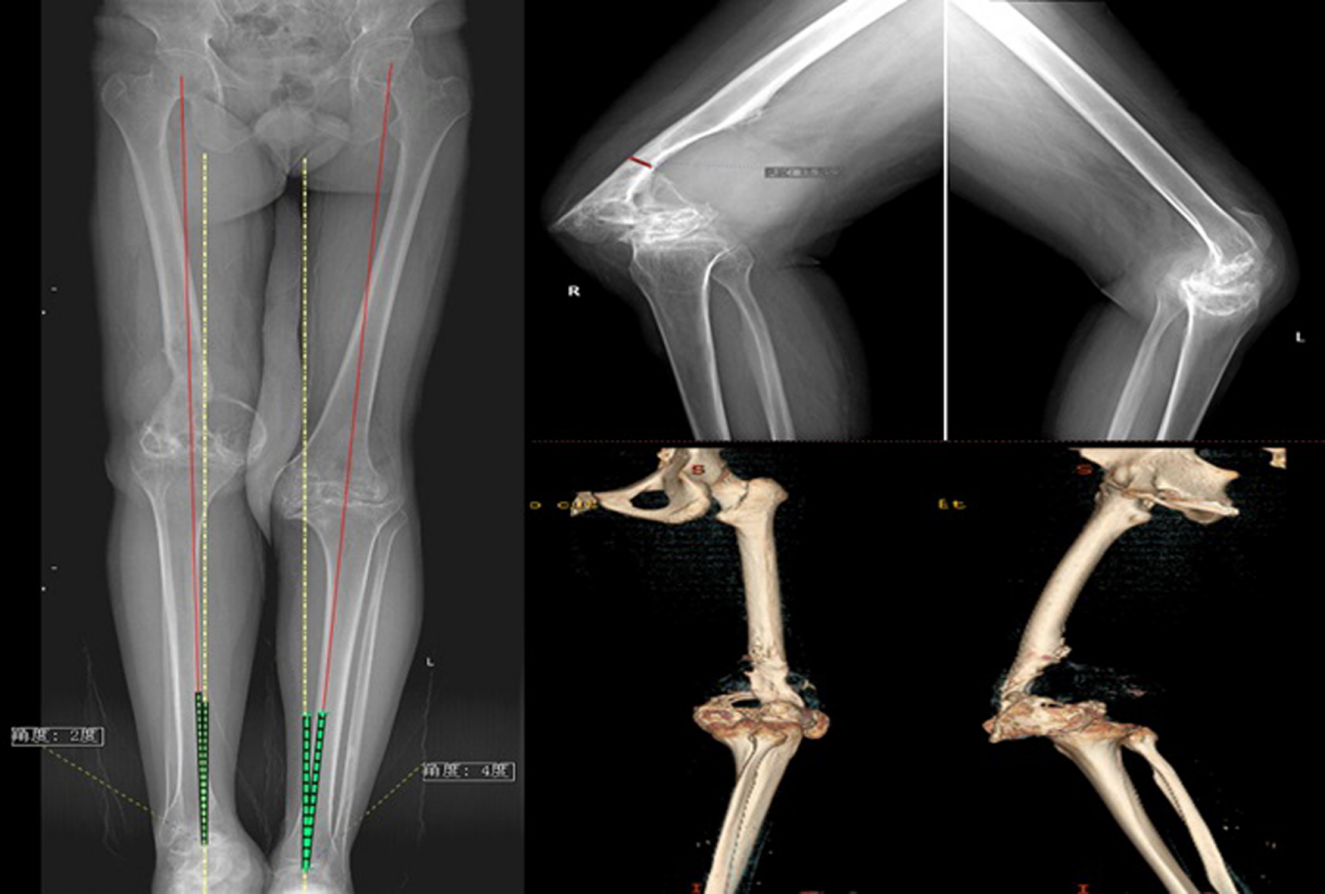
Preoperative roentgenograms and CT reconstruction of hemophilic arthropathy of the knee.

**Figure 2:**
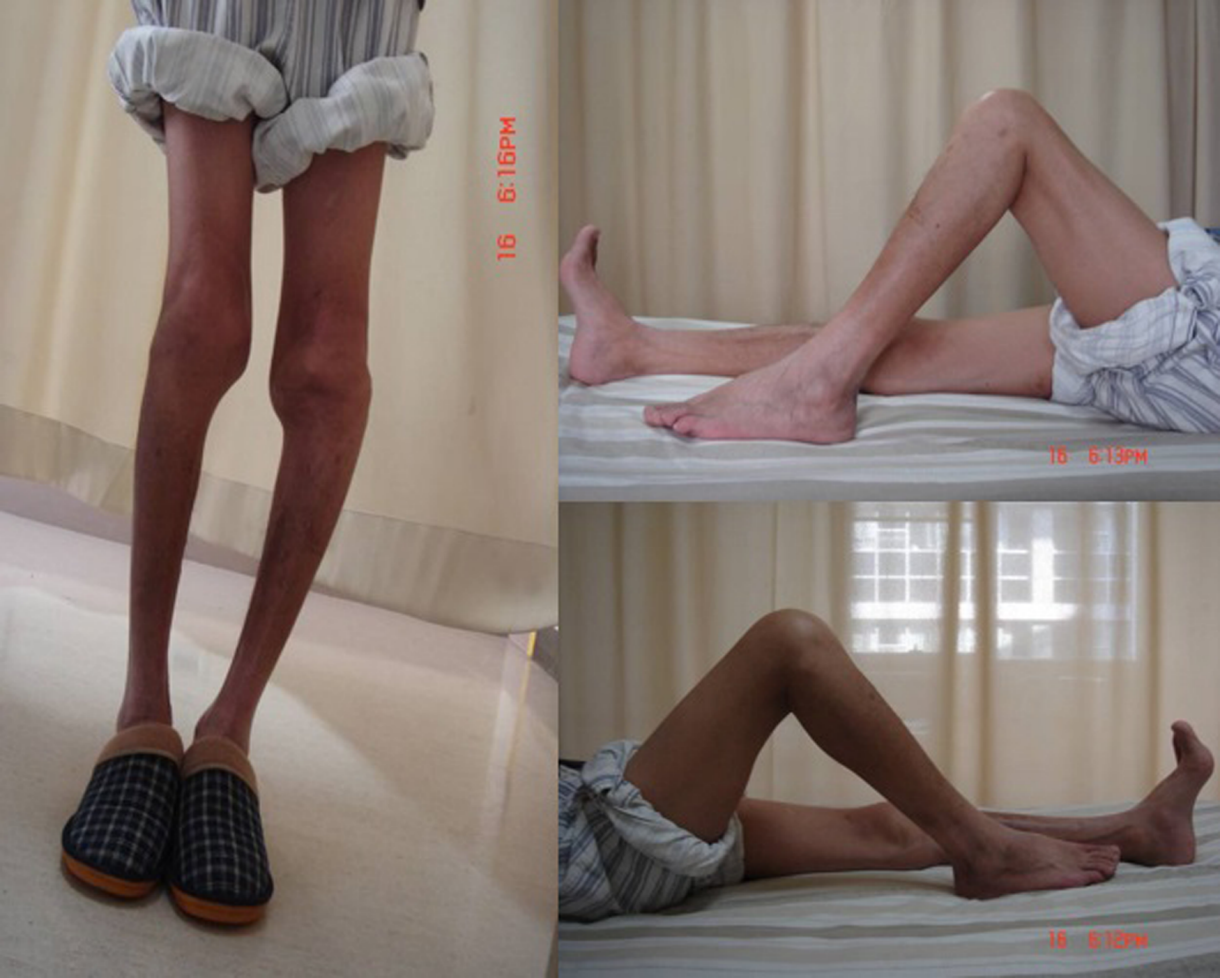
Clinical photograph showing preoperative deformity in patients with hemophilia.

**Figure 3:**
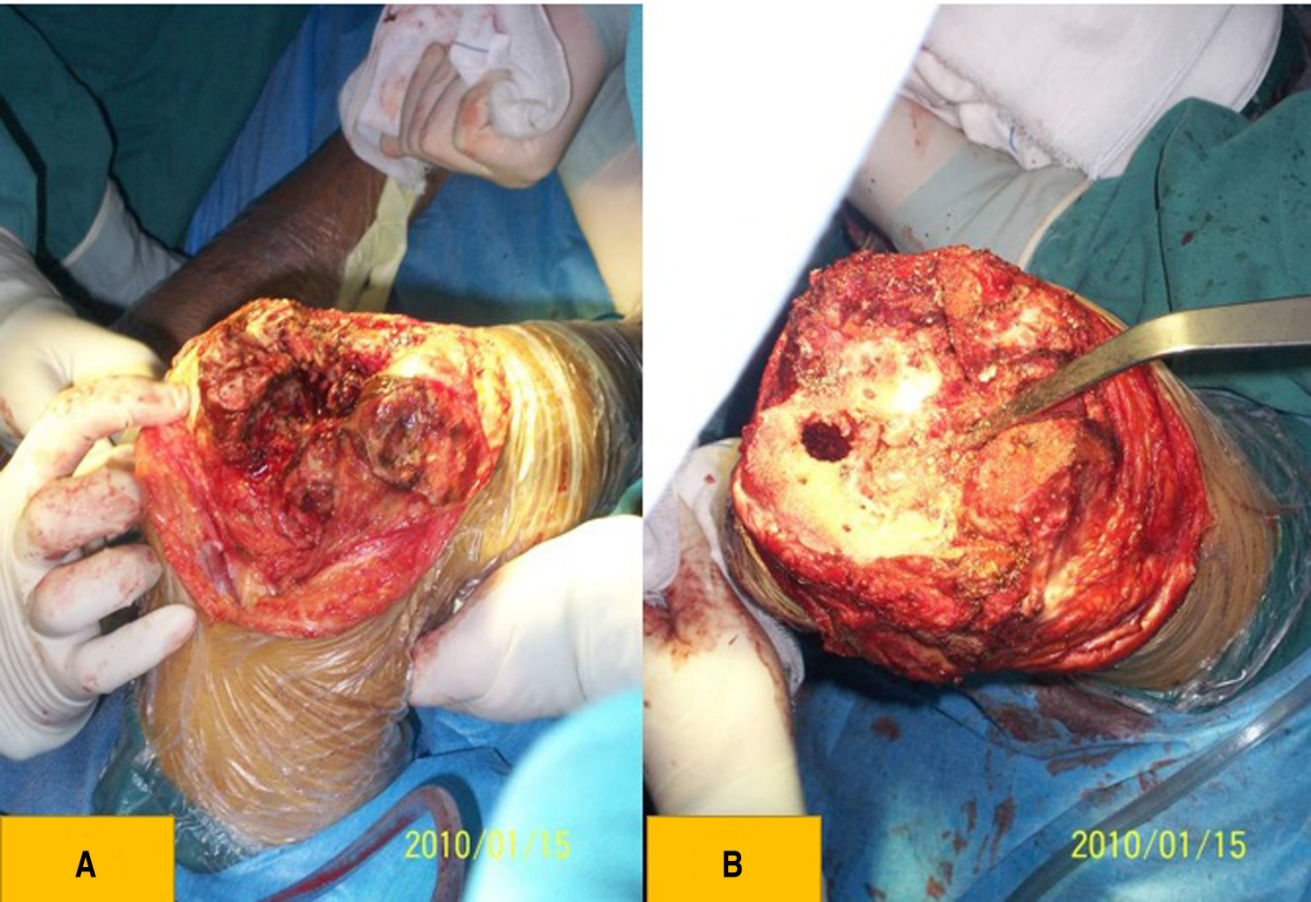
(**A**) clinical photograph showing that the bone structure was dysplastic, the patella was thin, and the femur was golf-club like and severely worn. (**B**) Clinical photograph showing tibial plateau dysplasia and bone defects.

The inclusion criteria were as follows: (1) hemophilia patients with stiff knees treated with TKA between September 2007 and June 2018, (2) Arnold–Hilgartner stage IV and V hemophilic arthropathy (8) (Figure 1), and (3) gait disorders and dysfunction due to severe pain and limited range of motion (ROM) as well as ineffective conservative treatment (Figure 2). The exclusion criteria were as follows: (1) lack of follow-up or incomplete radiological data and (2) other simultaneous surgical interventions, including total hip replacement, ankle replacement, or elbow replacement.

### Patient data and study design

During this period, 85 patients (94 knees) with rigid hemophilia were treated at our hospital. Six patients (seven knees) were excluded due to loss to follow-up by 4 years, five patients (five knees) underwent total hip or ankle replacement during the same period, and the imaging data of three patients (four knees) were incomplete. Finally, 71 patients (78 knees) with hemophilia were included in this study. All patients were male, their mean age at the time of surgery was 38.4. ± 7.9 years (range: 21–63 years), and the mean follow-up time was 8.7 years. General patient information is shown in Table 1. All patients provided informed consent, and the study design was approved by the ethics committee of The First Affiliated Hospital of Shandong First Medical University (2022-S523).

**Table 1:** Patient demographics.

Patient demographics	
Number of patients (*n*)	71
Number of knees (*n*)	78
Age (y)^a^ (range)	38.4 ± 7.9, (21–63)
20–39 (%)	23 (32.4)
30–39 (%)	25 (35.2)
40–49 (%)	18 (25.3)
50–59 (%)	4 (5.6)
60–69 (%)	1 (1.4)
Follow-up period (y)^a^ (ranges)	8.7 ± 1.97, (4.3–15.2)
Hemophilia type (A/B)	62/9
ROM (<50°/50–90°)	34/37
HIV/HCV	0/6
Factor VIII inhibitor positive (%)	5 (7.0)

^a^ Values are presented as the mean ± standard deviation.

ROM, range of motion; HIV, human immunodeficiency virus; HCV, hepatitis C virus.

### Perioperative management

All patients were treated based on the World Hemophilia Foundation guidelines, and treatments were adjusted based on clinical experience and economic status. Under normal circumstances, the concentration of coagulation factors was maintained at approximately 100% on the day of surgery, 80% in the first 3 postoperative days, 60% on days 4–6 after surgery, 40% on days 7–14 after surgery, and gradually decreased to 20%–30% thereafter. In addition to ensuring coagulation factor infusion, blood preparation was routinely performed preoperatively, and the dosage of coagulation factors was adjusted based on intraoperative blood loss and hemoglobin monitoring.

### Operative technique

Standard radiographs (AP view, lateral view, and long-leg standing radiographs) were taken before surgery. Bone destruction was visualized on 3D CT bone reconstruction. Patients received intravenous tranexamic acid (TXA) 10 min before the skin incision (15 mg/kg) and topical TXA (1 g) was applied intra-articularly to each operated joint after articular capsule closure. We regularly administered prophylactic antibiotics *via* intravenous injection (cefazolin 2.0 g) 30 min before surgery and once afterward. Suction drains were not used to reduce postoperative bleeding and prevent infection. Intraoperative periarticular injections of ropivacaine and postoperative oral tramadol (50 mg twice daily) were administered to relieve pain. Surgeries were performed under general anesthesia with a tourniquet, and the procedure was similar to that of routine TKA (Figure 3). Most patients underwent the medial parapatellar approach (Table 2). The V–Y quadriceps femoris approach was used for 18 (23.1%) stiff knee joints with exposure and flexion difficulties. If flexion deformity could not be completely corrected during surgery, plaster braking and skin traction were implemented postoperatively. Different types of prostheses were used based on the degree of flexion deformity, ligament stability, and bone defects (Table 2).

**Table 2:** Surgical procedures.

Surgical procedures	Severe group (*n* = 34)	Moderate group (*n* = 44)	*p*-value
Medial parapatellar approach	21 (61.7)	39 (88.6)	<0.001
V–Y quadricepsplastya	13 (38.3)	5 (11.4)	
PS prosthesis	16 (47.1)	36 (81.8)	<0.001
VVC prosthesis	15 (44.1)	7 (15.9)	
Hinged prosthesis	3 (8.8)	1 (2.2)	
Patellar resurfacing	26 (76.4)	34 (77.2)	0.893
Patellar non-resurfacing	8 (23.6)	10 (22.8)	

Data are presented as numbers and percentages in the parentheses.

PS, posterior stabilized; VVC, varus-valgus constrained.

### Postoperative rehabilitation

None of the patients received anticoagulants. Physical methods, such as ankle pump and lower-limb elastic bandage, were used to prevent lower-limb deep venous thrombosis, and patients were instructed to perform straight leg lifting and ankle flexion as well as extension exercises. Lower-limb continuous-passive-motion training and lower-limb functional exercises assisted by walking aids were gradually performed. Personalized family rehabilitation and factor replacement programs were provided after discharge.

### Clinical evaluation

Patients were followed up by telephone or at the outpatient clinic at 3 months, 6 months, and 1 year postoperatively. Patients were followed up annually thereafter. The Knee Society score (KSS) and KSS function score were used to evaluate knee function, the Short Form-36 score (SF-36) was used to evaluate quality of life, and the visual analysis scale (VAS) was used to evaluate the degree of pain. According to the KKS functional score, the overall results of greater than 85 points were regarded as excellent, those of 70–84 points as good, those of 60–69 points as general, and those of less than 59 points as poor (9). Flexion contracture and ROM were measured using a long-arm goniometer (QK-JDC-1, China). The measurements were made based on the angle between the line from the greater trochanter to the lateral femoral condyle and the line from the lateral condyle to the tip of the lateral malleolus, centered on the most convex point of the lateral femoral condyle. Postoperative complications were recorded, and prosthesis survival rates at 5 and 10 years were evaluated.

Most previous studies defined preoperative stiffness as when the ROM was less than 50°, whereas other studies used 90° to define preoperative stiffness (10, 11). Therefore, we divided the subjects into two groups: a severe group (preoperative ROM, 50°; 34 knees) and a moderate group (preoperative ROM, 50°—90°; 44 knees) (Table 4). Clinical function and complication rates were compared between the two groups.

### Statistical analysis

SPSS statistical software (version 26.0; IBM SPSS, Armonk, NY, USA) was used for statistical analyses. An independent-samples *t*-test was used to compare preoperative and postoperative KSS, SF-36, VAS, ROM, and flexion deformity scores. Student's *t*-test was used to compare the postoperative flexion deformity, ROM, and KSS scores between the severe and moderate groups. To determine whether our sample had sufficient power to detect a significant difference, we performed *post hoc* power analysis with a significance level for Student's *t*-tests and independent *t*-tests set to an alpha of .05. Power >80% was considered adequate, and all significantly different variables met the criteria.

## Results

### Clinical evaluation

The mean follow-up time was 8.7 ± 1.97 years. At the final follow-up, flexion contracture improved from 23.2 ± 10.8° before surgery to 5.9 ± 7.5° at final follow-up (*p* < 0.001) (Table 3), the KSS score increased from 31.4 ± 12.4 to 74.9 ± 11.5 (*p* < 0.001), and the KSS function score increased from 37.6 ± 9.3 to 81.4 ± 12.8(*p* < 0.001). The mean ROM increased from 54.6 ± 32.6° before surgery to 80.9 ± 34.5° after surgery (*p* < 0.001). The average VAS score decreased from 8.5 points before surgery to 2.1 points after surgery. The SF-36 physical and mental scores also significantly improved, and the differences before and after surgery were statistically significant; According to the KSS function scores, the overall result was excellent in 62.8% (49/78) and good in 32.0% (25/78), respectively.

**Table 3:** Clinical outcomes between the preoperative stage and final follow-up.

Functional outcome	Preoperative	Final follow-up	*p*-value
Range of motion (◦)	54.6 ± 32.6	80.9 ± 34.5	<0.001
Flexion contracture (◦)	23.2 ± 10.8	5.9 ± 7.5	<0.001
KSS	31.4 ± 12.4	74.9 ± 11.5	<0.001
KSS function	37.6 ± 9.3	81.4 ± 12.8	<0.001
SF-36 physical score	27.3 ± 13.9	69.13 ± 14.2	<0.001
SF-36 mental score	31.48 ± 6.53	60.4 ± 7.12	<0.001
Overall result:			
Excellent, *n* (%)	–	49 (62.8)	<0.001
Good, *n* (%)	–	25 (32.0)	<0.001
Fair, *n* (%)	59 (75.6)	2 (2.5)	<0.001
Poor, *n* (%)	19 (24.3)	2 (2.5)	<0.001

KSS, American Knee Society score; SF-36, Short Form-36 score.

The mean ROM in the severe group increased from 30.7 ± 18.7° preoperatively to 70.5 ± 28.3° postoperatively (*p* < 0.001). The mean flexion contracture decreased from 27.3 ± 10.8° to 6.4 ± 12.0° (*p* < 0.001). The mean KSS improved from 27.0 ± 7.8 to 68.3 ± 9.6 (*p* < 0.001). The mean ROM in the moderate group improved from 84.3 ± 22.7 to 92.9 ± 28.8 (*p* < 0.001). The mean flexion contracture decreased from 12.8 ± 11.0° to 4.8 ± 5.0° (*p* < 0.001). The mean KSS improved from 41.3 ± 11.5 to 81.3 ± 12.2 (*p* < 0.001). The severe group had worse postoperative ROM and functional scores than the moderate group, and recurrent postoperative stiffness was more common than in the moderate group (8.8% vs. 2.0%)(Table 4). Furthermore, the severe group used varus-valgus constrained or hinged prostheses more frequently (52.8% vs. 18.1%) and had more complications (18.9% vs. 9.0%) than the moderate group.

**Table 4:** Clinical outcomes between the severe and moderate groups.

	Severe group (*n* = 34)	Moderate group (*n* = 44)	*p*-value
Flexion contracture (°)			
Preoperative	27.3 ± 10.8	12.8 ± 11.0	<0.001
Final follow-up	6.4 ± 12.0	4.8 ± 5.0	<0.426
Range of motion (°)			
Preoperative	30.7 ± 18.7	84.3 ± 22.7	<0.001
Last follow-up	70.5 ± 28.3	92.9 ± 28.8	<0.001
KSS function (points)			
Preoperative	27.0 ± 7.8	41.3 ± 11.5	<0.001
Last follow-up	68.3 ± 9.6	81.3 ± 12.2	<0.001

KSS, American Knee Society score.

### Radiographic evaluation

**Figure 4:**
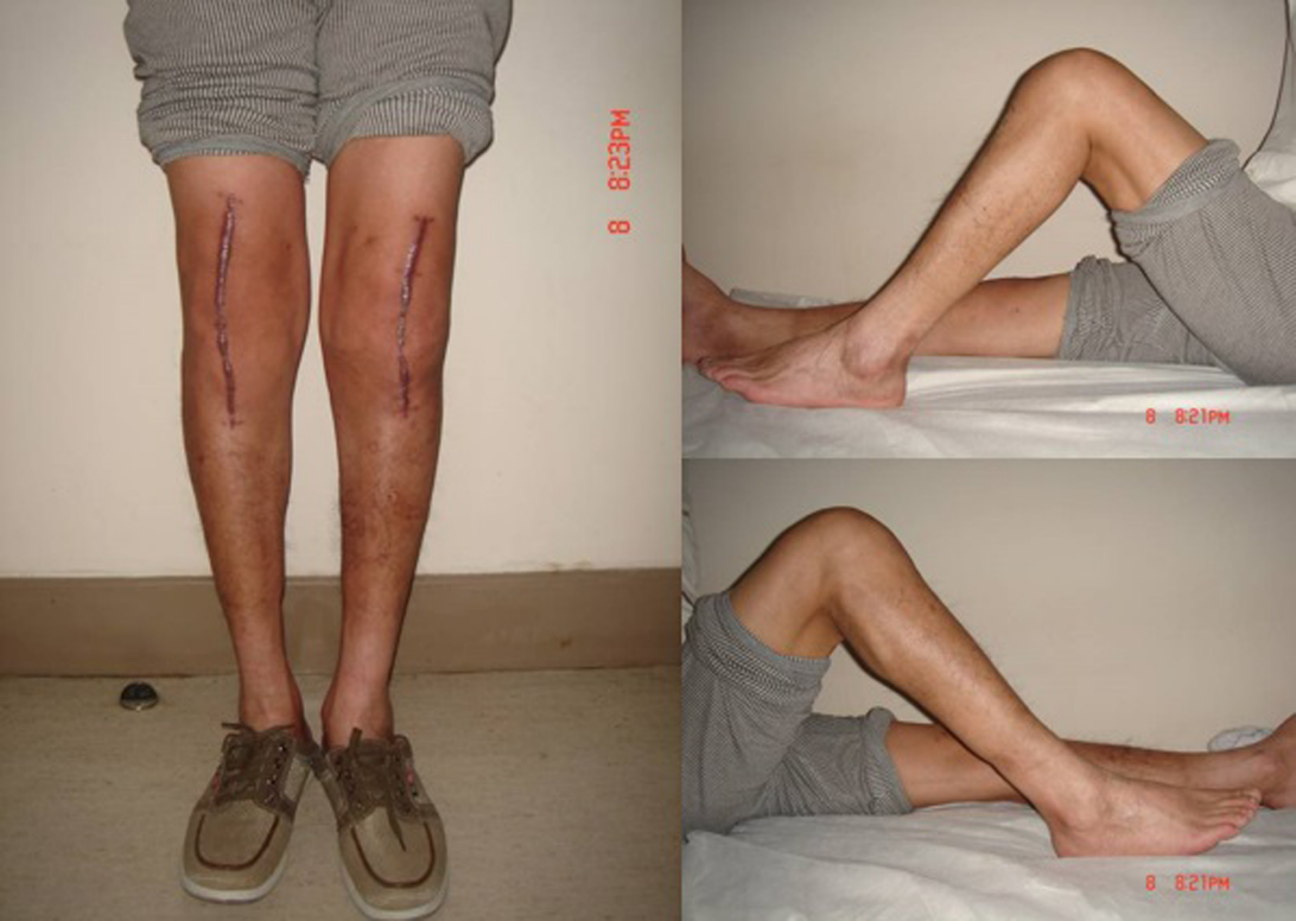
The deformity was completely corrected 6 weeks after surgery.

**Figure 5:**
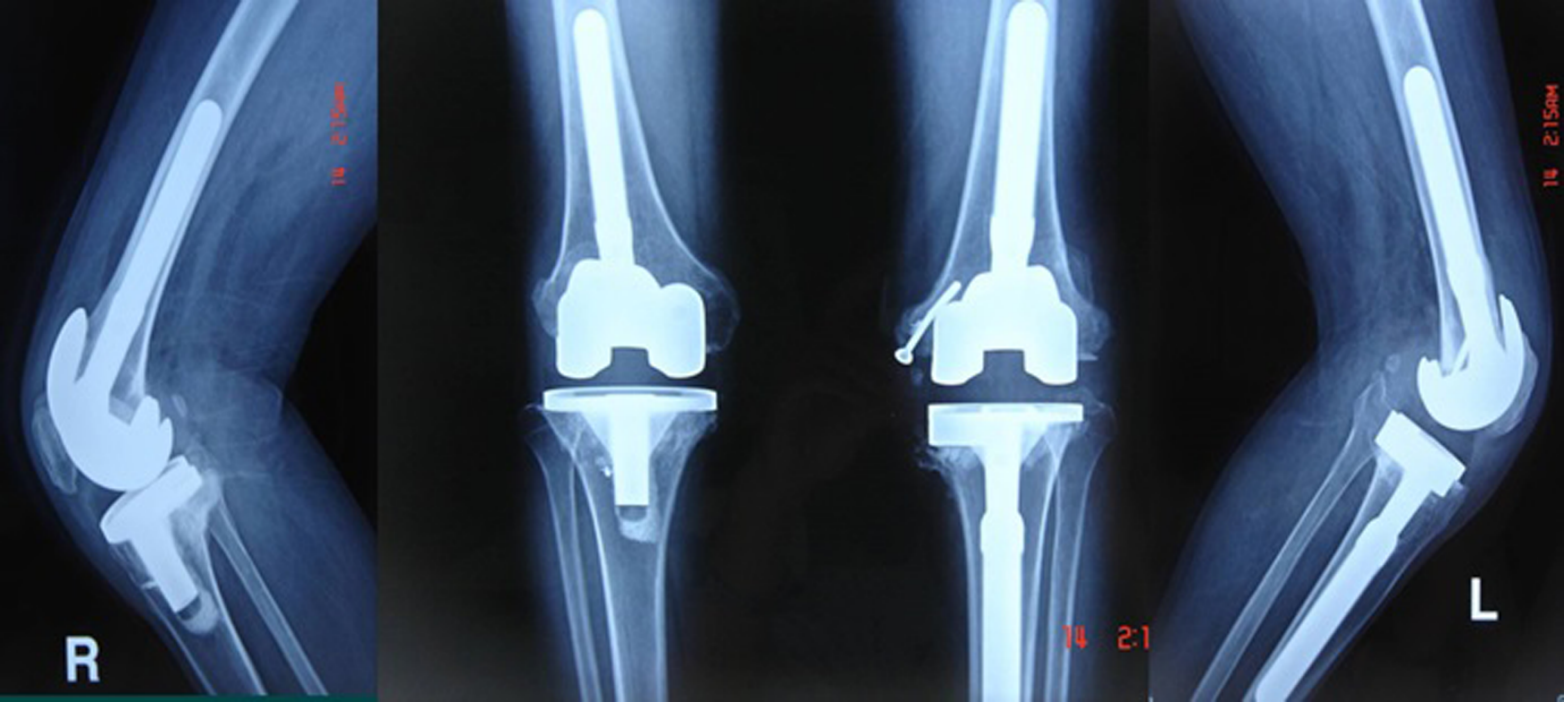
Radiograph of the anterior and lateral post-TKA positions after TKA, indicating that the position of the prosthesis was favorable.

The radiograph of the knee joint after prosthetic position evaluation demonstrated that the prosthetic position was favorable, and the force line of the lower limbs was completely restored (Figures 4, 5). One patient (one knee) had a light transmission line around the femoral prosthesis 13 months after the operation, and it was considered a periprosthetic infection; no loosening, sinking, or osteolysis of the prosthesis was found in other cases.

### Complications and survival rate

Postoperative complications occurred in 11 knees (14.1%) (Table 5). The rehospitalization rate 3 months after TKA was 10.2% (8/78 knees). The reasons for rehospitalization were joint hemorrhage, joint stiffness, superficial infection, and skin necrosis. Two knees (2.5%) underwent TKA due to periprosthetic joint infection 2 and 2.4 years after surgery. With prosthetic revision as the endpoint, the 5- and 10-year survival rates of the prosthesis were 98.5% and 93.7%, respectively.

**Table 5:** Postoperative complications between the severe and moderate groups.

	Severe group (*n* = 34)	Moderate group (*n* = 44)	*p*-value	Treatment
Complications, *n* (%)	7 (18.9)	4 (9.0)	<0.001	
Hemarthrosis, *n*	2	1		Incision and drainage of hematoma
Stiffness, *n*	3	1		Manipulation under anesthesia
Superficial infection, *n*	1	0		Anti-infection
Skin necrosis, *n*	0	1		Flap transplantation
PJI, *n*	1	1		Two-stage revision TKA

PJI, periprosthetic joint infection.

## Discussion

The most important finding of this study is that the long-term, clinical TKA results for the treatment of rigid hemophilic knee arthritis were satisfactory, thus indicating that TKA can significantly improve the postoperative function of patients, correct joint flexion deformity, and improve quality of life. Preoperative stiffness has been proven to be an important factor affecting the post-TKA function of patients with osteoarthritis. Few studies have examined the influence of stiffness on the clinical outcomes and complication rates of patients with hemophilia. Alpaslan et al. followed up 14 patients (21 knees) with hemophilia for 5.7 years and found that the degree of preoperative flexion spasm determined the clinical function of TKA in the treatment of hemophilic arthropathy (12). Straus et al. followed up 21 patients (23 knees) with hemophilia who had had a preoperative ROM ≤ 50° for 8.3 years, and their joint ROM increased from 26.7° before surgery to 73.0° after surgery. Flexion contracture decreased from 21.7° to 8.3°. The KSS score increased from 22.9 to 72.9 (5). Our study found that although the severe group (preoperative ROM: <50°) had more severe stiffness than the moderate group (preoperative ROM: 50–90°), it exhibited a greater ROM increase; however, the final joint ROM and clinical score in the severe group were poor, and the incidence of complications was higher than that in the moderate group. Even in the case of severe preoperative stiffness, TKA can effectively improve the joint mobility and functional scores of patients with hemophilia; however, surgeons should be aware of the poor clinical outcomes of patients with severe preoperative stiffness.

Long-term survival following TKA in nonhemophilic patients is well reported; higher rates of sterile mechanical failure of implants and shorter survival periods have been reported following TKA, especially in patients ≤50 years of age. Meanwhile, studies evaluating the post-TKA long-term survival of patients with hemophilia are limited (7, 13, 14). Generally, the implant survival rate of patients with hemophilia is considered to be lower than that of patients without hemophilia (15). Possible reasons include a younger age at the time of surgery, high activity, heavy wear of the prosthesis, and a high incidence of postoperative infection. In recent years, with the development of coagulation factor replacement therapy and improvements in prosthetic design, the survival rate of prostheses in patients with hemophilia after joint replacement has greatly improved. With prosthetic revision as the endpoint, the 10-year survival rate of prostheses in patients with hemophilia can exceed 80% (7). In this study, 71 patients (78 knees) were followed for an average of 8.7 years. The prosthesis survival rate was 98.5% at 10 years after surgery, a result that is superior to that reported in previous studies.

The post-TKA infection rate of patients with hemophilia is reportedly 6.5%–16%, which is significantly higher than that of patients without hemophilia (16). The reasons for the high infection rate may include combined hepatitis C or immunodeficiency virus infection as well as bacteremia infection during the import of coagulation factors (1, 12). In this study, two patients developed periprosthetic joint infection, which was diagnosed 4.8 and 8.5 years after surgery, with an incidence of 2.5%. Although no definite etiology related to post-TKA periprosthetic infection in hemophilia exists, most patients undergo factor replacement on demand, thus potentially increasing the risk of bacterial contamination through the puncture point. Therefore, for hemophilia patients, more emphasis on aseptic surgery and the prophylactic application of antibiotics before invasive operation can still reduce the incidence of infection (10).

The post-TKA incidence of hemarthrosis in patients with hemophilia is reportedly significantly higher than that in patients without hemophilia (16). Studies have revealed that as the most common complication of hemophilia, the incidence of joint hemorrhage ranges from 12.5% to 25.6% (11). The main reasons include a low concentration of coagulation factors, the presence of coagulation factor inhibitors, synovial hemorrhage, and improper postoperative functional exercise (16). The risk of bleeding was highest within 1 week after surgery. Adequate replacement of coagulation factors and complete removal of the synovium during surgery are important measures for reducing bleeding. Mortazavi et al. (1) suggested that injecting tranexamic acid into the articular cavity and closely suturing the wound after surgery potentially reduce postoperative bleeding. All patients in this group underwent coagulation factor replacement before surgery. The synovium was completely removed, and tranexamic acid was injected into the articular cavity intraoperatively. In the present study, the incidence of postoperative joint bleeding was 5.1%, which is lower than the previously reported incidence of hemophilia. We believe that careful surgical techniques combined with specific rehabilitation and appropriate factor replacement schemes can reduce the incidence of postoperative joint bleeding.

This study had certain limitations. First, this was a retrospective study, with non-flexible knees constituting the control group. Considering the rare incidence of hemophilia, it is difficult to conduct such a prospective study in a single institution. Second, the 5-year follow-up time for some patients was relatively short. Hence, we could not determine the occurrence of late complications, such as aseptic loosening of the prosthesis or late prosthetic joint infection, which potentially develops over a long period after surgery. Third, all surgeries were performed by two senior surgeons, and different surgeons may be associated with an increased risk of infection or failure. Moreover, we used different implants according to the patient's condition. Fourth, we did not evaluate radiological outcomes for lower limb alignment. Finally, the age range of the participants in this study was large (21–63 years), and we did not investigate whether clinical efficacy and complications were associated with age in our analysis. Since TKA for hemophilia is a technically demanding procedure, we reviewed 78 knees from 71 hemophilia patients, which is a large number for a single institution. Despite the aforementioned limitations, the strengths of this study include the successful long-term results and good prosthesis survival, which has not been previously reported in a large-scale population from a single research institute.

## Conclusion

TKA yields satisfactory mid-to-long-term results in the treatment of rigid hemophilic arthropathy, with significant improvements in function and pain relief. Furthermore, severely stiff knee joints have worse clinical outcomes and more complications than do moderately stiff knee joints.

## Data Availability

The raw data supporting the conclusions of this article will be made available by the authors, without undue reservation.
